# Enhanced in vitro model of the CSF dynamics

**DOI:** 10.1186/s12987-019-0131-z

**Published:** 2019-04-29

**Authors:** Anne Benninghaus, Olivier Balédent, Armelle Lokossou, Carlos Castelar, Steffen Leonhardt, Klaus Radermacher

**Affiliations:** 1 Chair of Medical Engineering, Helmholtz-Institute for Biomedical Engineering, RWTH Aachen University, Pauwelsstraße 20, 52074 Aachen, Germany; 2 Department of Image Processing, University Hospital, E.A 7516, CHIMERE, Jules Verne University of Picardy, 80054 Amiens cedex, France; 3 Chair for Medical Information Technology, Helmholtz-Institute for Biomedical Engineering, RWTH Aachen University, Pauwelsstraße 20, 52074 Aachen, Germany

**Keywords:** In vitro modeling, CSF dynamics, Compliance, NPH

## Abstract

**Background:**

Fluid dynamics of the craniospinal system are complex and still not completely understood. In vivo flow and pressure measurements of the cerebrospinal fluid (CSF) are limited. Whereas in silico modeling can be an adequate pathway for parameter studies, in vitro modeling of the craniospinal system is essential for testing and evaluation of therapeutic measures associated with innovative implants relating to, for example, normal pressure hydrocephalus and other fluid disorders. Previously-reported in vitro models focused on the investigation of only one hypothesis of the fluid dynamics rather than developing a modular set-up to allow changes in focus of the investigation. The aim of this study is to present an enhanced and validated in vitro model of the CSF system which enables the future embedding of implants, the validation of in silico models or phase-contrast magnetic resonance imaging (PC-MRI) measurements and a variety of sensitivity analyses regarding pathological behavior, such as reduced CSF compliances, higher resistances or altered blood dynamics.

**Methods:**

The in vitro model consists of a ventricular system which is connected via the aqueduct to the cranial and spinal subarachnoid spaces. Two compliance chambers are integrated to cushion the arteriovenous blood flow generated by a cam plate unit enabling the modeling of patient specific flow dynamics. The CSF dynamics are monitored using three cranial pressure sensors and a spinal ultrasound flow meter. Measurements of the in vitro spinal flow were compared to cervical flow data recorded with PC-MRI from nine healthy young volunteers, and pressure measurements were compared to the literature values reported for intracranial pressure (ICP) to validate the newly developed in vitro model.

**Results:**

The maximum spinal CSF flow recorded in the in vitro simulation was 133.60 ml/min in the caudal direction and 68.01 ml/min in the cranial direction, whereas the PC-MRI flow data of the subjects showed 122.82 ml/min in the caudal and 77.86 ml/min in the cranial direction. In addition, the mean ICP (in vitro) was 12.68 mmHg and the pressure wave amplitude, 4.86 mmHg, which is in the physiological range.

**Conclusions:**

The in vitro pressure values were in the physiological range. The amplitudes of the flow results were in good agreement with PC-MRI data of young and healthy volunteers. However, the maximum cranial flow in the in vitro model occurred earlier than in the PC-MRI data, which might be due to a lack of an in vitro dynamic compliance. Implementing dynamic compliances and related sensitivity analyses are major aspects of our ongoing research.

## Introduction

The CSF is an aqueous fluid containing small concentrations of various proteins, glucose and electrolytes which surrounds the central nervous system and, consequently, serves as a ‘lymphatic’ system and a mechanical shock absorber. The rates of CSF production and absorption are usually in equilibrium. However, the exact locations for production and absorption are still being discussed [1, 2]. In addition, the fluid dynamics of the craniospinal system are determined primarily by the rapid in-and outflow of blood to the cranial compartment, driving the fluid to the more distensible spinal compartment in systole and returning to the cranium in diastole [3–6].

If the CSF dynamics are disturbed, due to aging or changes in blood dynamics, compliance, production and absorption, or resistance, and pathological conditions can be observed by measuring abnormal intracranial pressure (ICP) or CSF flows. Normal pressure hydrocephalus (NPH) is a pathological condition, which predominantly occurs in the elderly (65 years+), and results in a pathological enlargement of the brain ventricles without an attendant rise in mean ICP. The symptoms of gait ataxia, urine incontinence and dementia, which can occur in the elderly, make the diagnosis difficult [7]. According to Hakim et al. up to 10% of all demented patients might be suffering from NPH [8]. However, the pathogenesis is still not understood and, therefore, effective therapy for NPH patients is still lacking. Many hypotheses suggest that biomechanical alterations due to aging upset the craniospinal dynamics and, thereby, play an important role in the formation of NPH [9–15].

There are different ways to investigate CSF dynamics and particularly the onset of NPH. Phase-contrast magnetic resonance imaging (PC-MRI) is an established tool to investigate the CSF or blood flow in vivo. A recent study on the accuracy of PC-MRI showed that the measuring error of a pulsatile flow is less than 10% [16]. On the one hand, in vivo data, such as flow measurements, provide information about the healthy and pathological conditions. On the other hand, this data is limited, and it is difficult to draw conclusions about the origin of the diseases. Furthermore, sensitivity analyses on the living organism are not possible, and the mechanical properties of the central nervous system tissue degenerate postmortem. Therefore, animal studies are often used to provide insights concerning issues such as absorption distribution or opening pressures [17]. In addition to the ethical aspects, transferability to humans must be taken into account, especially if hydrodynamics are considered, since the upright gait of humans differs fundamentally from the quadruped walk of most mammals. Moreover, the main knowledge about fluid mechanics originates from chemical, cellular or tissue aspects, although pathological conditions may only be derived from disturbed fluid mechanics.

Modeling (in silico or in vitro) the craniospinal system is an effective tool for analyzing the CSF system. There are varieties of in silico models which are commonly used for parameter examinations [15]. A distinction is made between computational fluid dynamics and lumped parameter models, which usually focus on a specific question. Lumped parameter models are often imaged by mechanical or electrical analogies but cannot map the spatial resolution flux distribution [18]. Computational fluid dynamic models calculate spatially resolved information of the system dynamics, such as pressure, flow or mass transport, but require high performance computing [19]. Hence, the hypothesis determines the appropriate kind of simulation. Nonetheless, there is no numerical tool for the entire CSF system and, additionally, simulation models cannot test implants.

In vitro models enable sensitivity analyses as well as the integration and testing of implants, such as shunt testing systems [20, 21]. Furthermore, there are a number of in vitro models for the craniospinal system extant, such as an artificial spinal canal [22, 23] or the modeling of cerebral vascular vessels [24]. In addition, two models have focused on the depiction of the craniospinal system as a whole. The model of Bouzerar et al. aims particularly at the investigation of the transmission of the blood pulsation to CSF dynamics and allows flow studies with altered hydrostatics to be made [25]. However, adjustable compliances and flow resistances were not considered. The second model by Bottan et al. focuses on the anatomically correct imaging of the cranial space with two adjustable compliance units, without the consideration of an attached spinal canal [26]. Thus, no hydrostatic investigations could be conducted.

Consequently, our goal was to design a model of the CSF dynamics which enables the investigation of its aging process and pathological transformation by conducting a variety of sensitivity analyses. We designed an in vitro model including brain parenchyma, cranial and spinal subarachnoid space (SAS), as well as adaptable compliances, blood pulsation and resistances to examine the dynamics. In contrast to in silico models of the CSF dynamics, our model also enables the future embedding and testing of alternative therapy methods. Moreover, the model can be used to validate simulation models. [27]

## Materials and methods

**Fig. 1:**
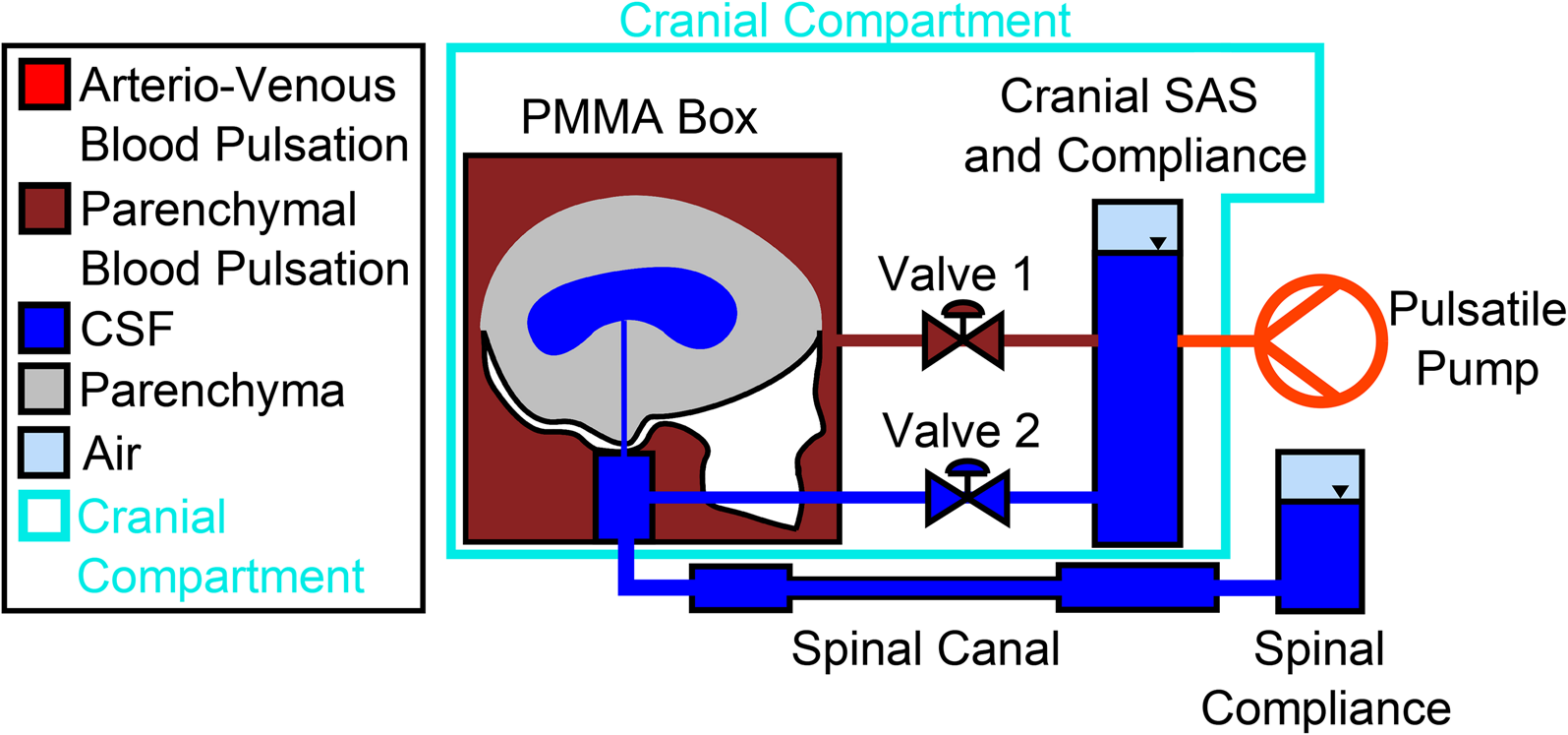
Schematic drawing of the experimental setup with a PMMA box containing the parenchyma (gray) with an enclosed ventricular system (blue), the cranial subarachnoid space and the spinal canal. The arteriovenous (AV) blood flow is reproduced by a pulsatile pump (red) connected to the cranial compliance chamber. Valve 1 adjusts the pulsation from the cranial SAS to the parenchyma, transmitted by the surrounding water in the box (dark red) and Valve 2 represents the resistance of the cranial SAS. Cranial and spinal compliance chambers are filled with air (light blue) in addition to the CSF (dark blue)

**Fig. 2:**
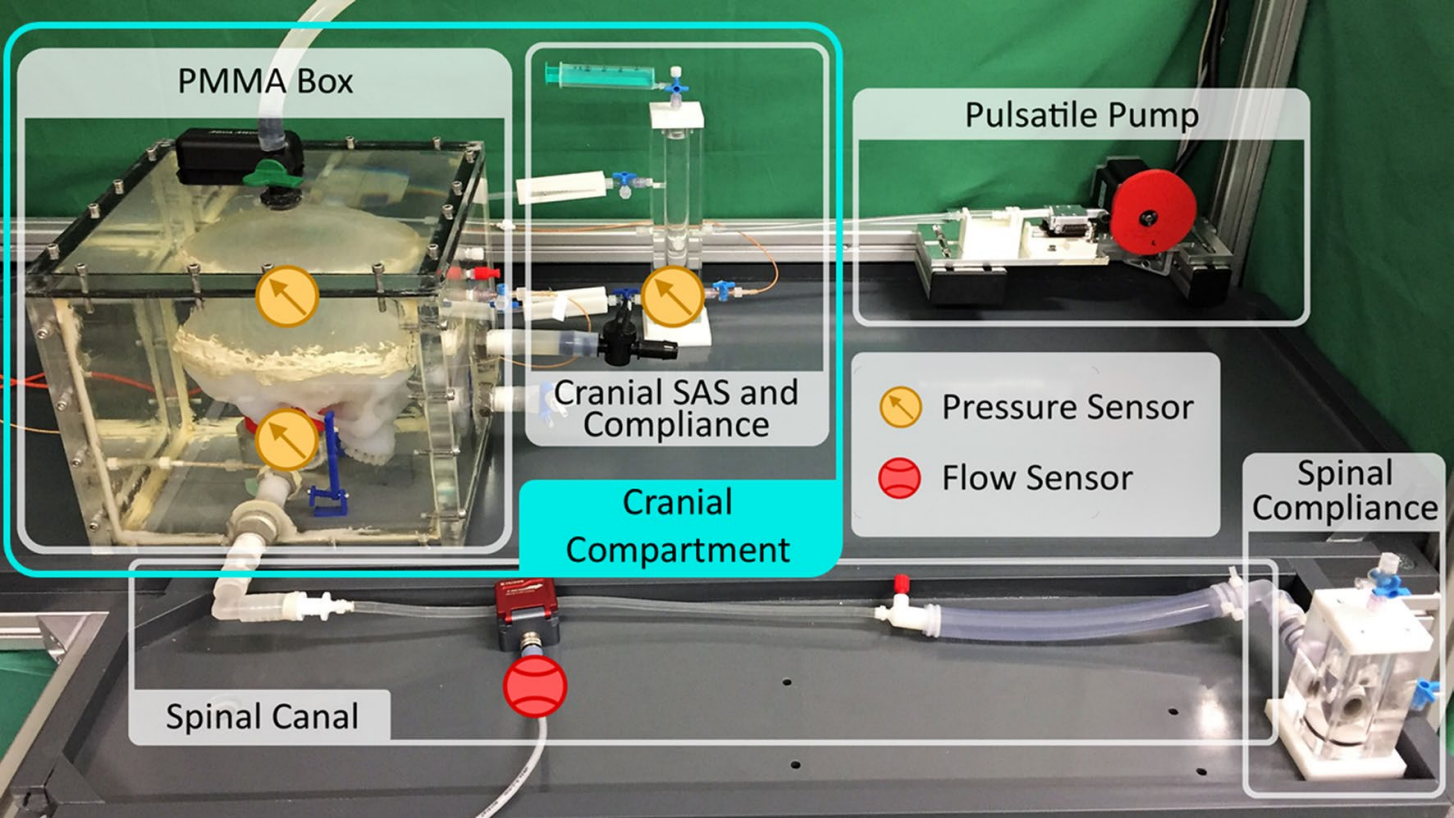
Experimental lab test bench. Three tip catheter pressure sensors (yellow) are placed inside the silicone parenchyma (measurement of ICP), the cistern and the compliance chamber of the cranial SAS. The ultrasound flow sensor (red) is attached at the upper part of the spinal canal (corresponding to the C2–C3 level)

The proposed phantom model design approach incorporates adjustable blood pulsation characteristics, cranial and spinal compliances, hydrostatics and flow resistance. These parameters can be varied to simulate physiological and pathological situations. The schematic set-up is shown in Fig. 1. There are three main CSF compartments connected to each other in the phantom model: The sealed polymethylmethacrylate (PMMA) box containing a parenchyma model with an enclosed ventricular system, the cranial SAS and the spinal canal. In addition, both cranial and spinal SAS are connected to separate compliance chambers. All compartments are filled with degassed water to represent the CSF. The corresponding lab test bench is shown in Fig. 2. As a first approach, production and absorption were neglected in the model due to the small flow volume compared to blood and CSF pulsations. A detailed list and part drawings of the components are available on request from the authors.

### Ventricular system and SAS

The PMMA box provides a rigid containment and accommodates a simplified ventricular system cast in a silicone brain (Sylgard 527, A&B Dielectric Silicone Gel, Dow Corning, Midland, Michigan). The parenchyma is glued to a plastic lower part of the skull, which is mounted to the bottom of the box. Whereas the skull prevents the silicone brain from ascending in the surrounding water due to density differences, it does not model a closed cranium. The ventricular system is modeled as one kidney-shaped cavity with a volume of 35.2 ml which combines both lateral and the third ventricle volumes. The cranial SAS is modeled by the cranial compliance chamber and the resistance (Valve 2). The pulsation of the pump is transmitted to the fluid in the PMMA box surrounding the parenchyma via the cranial compliance chamber and Valve 1. Water is a Newtonian fluid which is incompressible and transfers pulsatile energy directly into the CSF system. The flow from the cranial SAS into the PMMA box results in compression of the parenchyma and, thus, in a pulsating aqueductal flow. Furthermore, Valve 2 (Fig. 1) simulates an adjustable flow resistance within the cranial SAS. The other valve is situated between the cranial compliance chamber and the PMMA box and controls the pulsatile compression of the brain parenchyma (Fig. 1, Valve 1 (red)).

In a similar way to the cranial SAS, the flow resistance in the spinal canal plays an important role in the CSF dynamics. Therefore, the spinal canal is modeled by tubes with different diameters, which are connected to create an overall physiological hydraulic diameter varying from 5 to 15 mm (according to Loth et al. [28]). At the same time, the overall length of the spinal canal corresponds to a characteristic anatomical length and can be used to investigate the impact of hydrostatic changes on the CSF dynamics.

### Pulsatile pump

Vascular flow dynamics have an impact on the cranial and spinal CSF flow and pressure and are considered to majorly affect pathological conditions, such as NPH. During systole, 15% of the cardiac output is transferred to the brain via the *carotis interna* and *carotis vertebralis*. Subsequently, the blood leaves the cranial compartment through the veins [3, 9].

The arteries can expand and, therefore, flatten the pulsatile flow (Windkessel effect), whereas the veins can collapse and increase the cranial compliance. The subtraction of these two flows depicts the arteriovenous (AV) flow. Furthermore, the Monroe Kellie Doctrine states that the volume inside the cranium is invariable and remains the same throughout systole and diastole, because it is limited by the rigidity of the skull. By modeling the AV flow with a stroke volume (SV) of approximately $0.8\,\pm \,0.2\hbox { ml}$ into the cranium [14], the CSF shifts accordingly. Therefore, the change in blood volume directly affects the CSF dynamics in the cranial compartment. Boundary conditions in the spinal compartment differ from the cranial compartment. Although the spinal canal is also supplied with a pulsating blood flow, the spinal pulsation is much lower [29] and is, thus, negligible compared to the cranial pulsation.

**Fig. 3:**
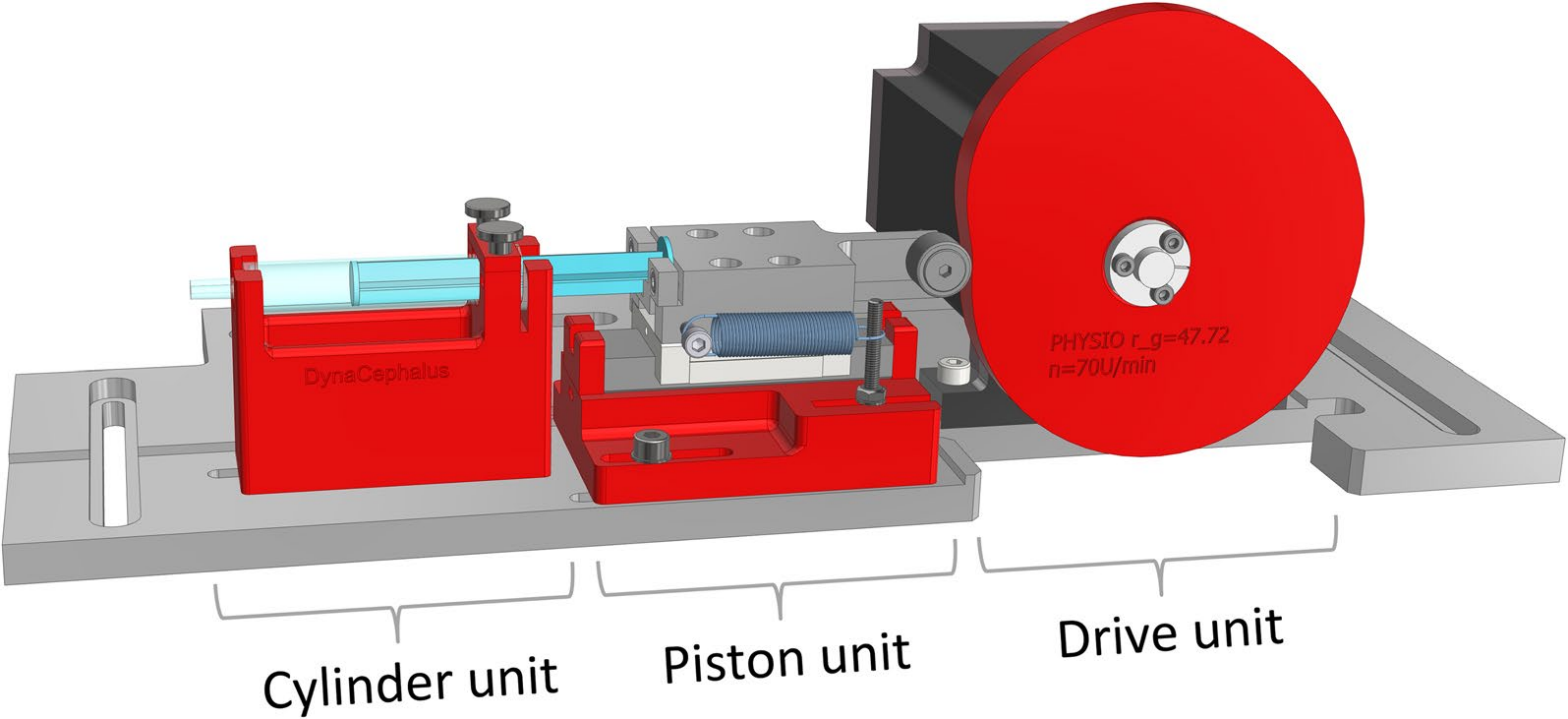
Cam plate driven piston pump. CAD model of the pulsatile pump, consisting of a cylinder unit, a piston unit and a drive unit with a patient-specific 3D-printed cam plate

**Fig. 4:**
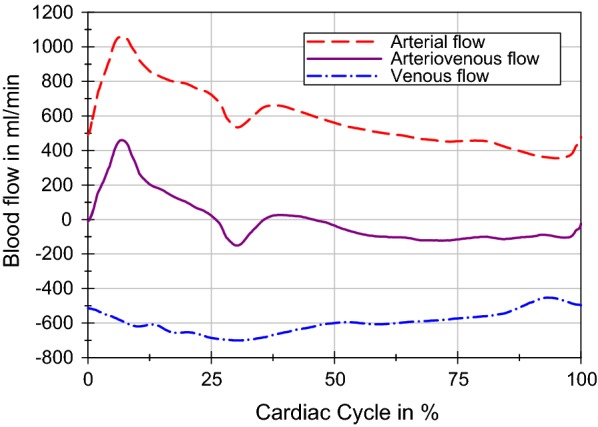
Pulsatile blood flow. The arterial inflow (red) and venous outflow (blue) add up to the AV flow-based (purple) PC-MRI measurements (data derived from ElSankari et al. [38])

We designed a cam plate-driven piston pump to reproduce the dynamic effects of the blood vessels on the CSF system. The assembly consists of three units: the drive unit, the piston and the cylinder (Fig. 3). The core piece is the drive unit, composed of a stepping motor and a controller (ST6018L3008-A and SMCI33-2, nanotec, Feldkirchen, Germany), and the cam disc. The piston unit, in combination with the cam roller and the defined outer cam contour, converts the rotary motion into correspondingly defined translational motion. The cylinder and the piston are parts of a common syringe (2 ml), which is connected to the cranial SAS through a polyvinyl chloride tube. The vascular effect on the CSF system can be changed easily by altering the disk contour according to the AV flow curves. The arterial and venous blood flow was measured at the C2–C3 level with PC-MRI. The venous outflow measured was shifted, so that the volume of the arterial inflow matched the venous outflow volume (Fig. 4). The AV flow is transferred to a cam disk using the hodograph transformation [30]. The resulting cam discs and the other red colored parts of the pump (Fig. 3) were manufactured using a FDM 3D printer (Ultimaker 3, Ultimaker B.V., Geldermansen, Netherlands). A connection of the PMMA box and the cranial SAS is established to model the variable effect of the parenchymal compression due to the AV blood pulsation by using another polyvinyl chloride tube and an adjustable valve (Valve 1 in Fig. 1).

### Compliance unit

Compliance is defined as the ratio of volume (V) to pressure (p) change and enables the system to accommodate a change of volume with an attendant pressure change [31].

1 $$\begin{aligned} C = \frac{dV}{dp} \end{aligned}$$

The compliance of the CSF system is based on vascular and tissue effects. Vascular effects on cranial compliance are associated mainly with collapsing veins [32]. However, the vascular compliance of arteries during the cardiac cycle (CC) also has an impact on the profile of blood pulsation [33]. Since the cranium is a rigid box, the vascular effects primarily affect the cranial compartment. In addition, the distal dural sac is the most compliant tissue in the CSF system [32]. Therefore, the division into a cranial and spinal compartment, adding up to the total compliance, is very common.

2 $$\begin{aligned} C_{total} = C_{cranial} + C_{spinal} \end{aligned}$$

However, there is still a debate concerning the distribution of the compliance [31, 34–36]. Consequently, two independent adjustable compliance units were connected to the model representing the cranial and the spinal compliant behavior. As a first approximation, these units, filled with water and air, model a static compliance. Since air can be described as an ideal gas, the following equation is used:

3 $$\begin{aligned} p_0 \cdot V_0^{\lambda } = p_1 \cdot V_1^{\lambda } \end{aligned}$$

with $\lambda =1.4$ indicating the isentropic exponent 0 the initial and 1 the resulting state. Combining Eqs. (1) and (3) and differentiating regarding pressure results in an equation for the compliance, which is only dependent on the pressure and the initial air volume in the container:

4 $$\begin{aligned} C = \frac{1}{\lambda } \root \lambda \of {p_0}\,V_0 (p_1)^{-\frac{1+\lambda }{\lambda }} \end{aligned}$$

Considering the pressure and its amplitude in the different compartments, the compliance can easily be adjusted by changing the initial volume of air. The setup parameters were chosen to simulate a physiological compliance in a supine position with a distribution of 0.31 ml/mmHg (27%) for the cranial and 0.84 ml/mmHg (68%) for the spinal compliance compartment (Table 1). Changing the position from supine to an upright position affects hydrostatic pressures and, thus, the compliance has to be taken into account concerning hydrostatic behavior.

**Table 1:** Compliance values and distributions in the in vitro model. Derived from Zweckberger et al. [60] and Tain et al. [31]

Compliance	Total	Cranial	Spinal
Value in ml/mmHg	1.15	0.31	0.84
Distribution in %	100	27	68

### Data Acquisition system—in vitro measurement

There are three tip catheter pressure sensors (NEUROVENT, Raumedic, Helmbrechts, Germany), measuring pressures between − 40 and 400 mmHg with an mean zero drift after 5 days of 0.6 mmHg [37]. The sensors were placed inside the silicone parenchyma (ICP), the cistern and the compliance chamber of the cranial SAS. In addition, an ultrasound flow meter (Sonoflow CO.55/060, Sonotec, Halle, Germany) was located at the beginning of the spinal canal (similar to C2–C3 level) to assess the cervical CSF flow in both directions (cranial/caudal, Fig. 2). The ultrasound technique enabled a contactless measurement, yet with an accuracy of 6 ml/min according to the manufacturer information. Due to the deviation of the flow value, the measurement was recorded over nine CCs. In addition, all sensors were connected to the computer data logging system NI cDAQ-9174 with the module NI 9237 for the pressure sensors and the module NI 9230 for the ultrasound flow sensor, which enabled the signal outputs to be recorded simultaneously and analyzed with the corresponding manufacturer software DIAdem (National Instruments, Austin, Texas, USA). All in vitro results were measured simulating 70 heart beats/min in a supine position. The pulsatile pump rotated twice before the recording starts to avoid a ramping effect.

### Data Acquisition system—in vivo measurement

In a previous study, CSF flow curves were calculated in nine healthy young adult volunteers on a 3 T machine using 2D fast cine PC-MRI pulse sequence with retrospective peripheral gating to reconstruct 32 frames covered the entire CC [3, 38]. The MRI parameters were as follows: two views per segment; flip angle: 20°; field-of-view (FOV): $14 \times 14$ mm$^2$; matrix: $256 \times 128$; slice thickness: 5 mm; one excitation. Velocity (encoding) sensitization was set to 5 cm/s. A sagittal scout view was used as a localizer. The selected acquisition plane was perpendicular to the presumed flow direction at the cervical level between the second and the third vertebra. Duration of the acquisition was around 2 min. Post processing was done with our homemade software [3].

**Fig. 5:**
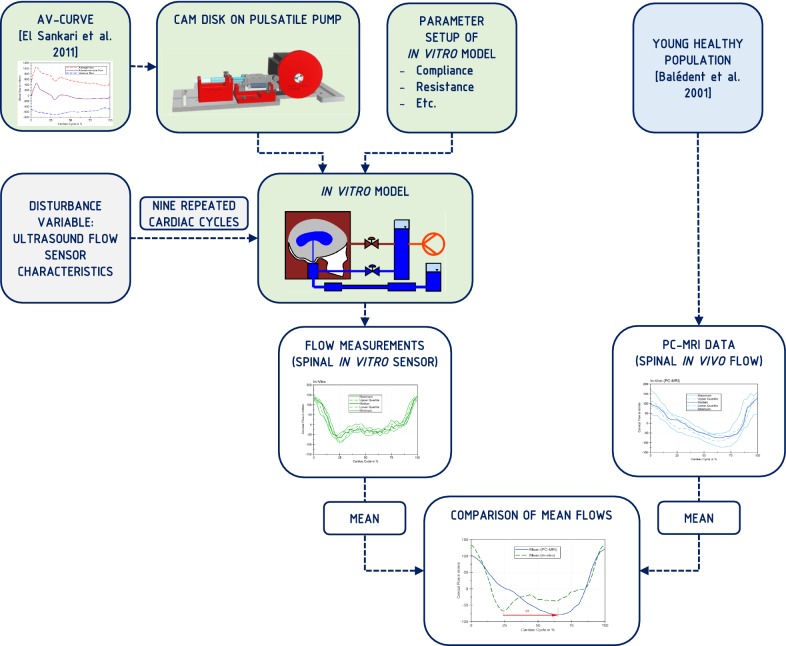
Flow chart of the flow measurement approach. Green shows steps connected to the in vitro model and blue to the in vivo data

The in vivo graphs (AV and CSF flow) are not synchronized in time, since the data was taken from different subjects. In vitro flow measurements were compared to the PC-MRI flow recordings. The time axes of the in vitro recordings correspond to the in vivo CSF flow data. The procedure of the flow measurements is shown in Fig. 5. Moreover, the in vitro ICP was compared with literature data and plotted from minimum to minimum.

## Results

### In vivo flow—PC-MRI

**Fig. 6:**
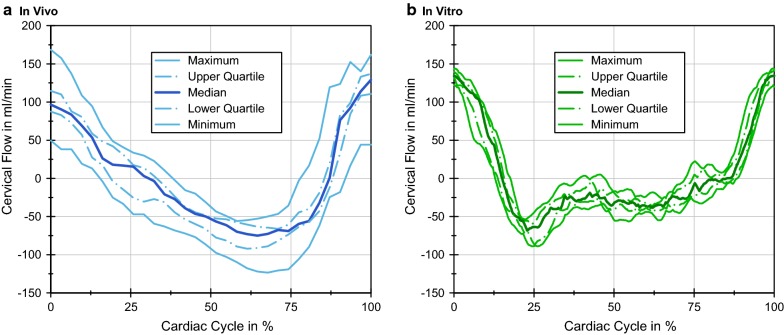
**a** PC-MRI (In vivo) measurements of the spinal CSF flow at the C2–C3 level. The range of the flow measurement and the median flow of nine young healthy volunteers is shown. **b** In vitro measurements of the spinal CSF flow at the C2–C3 level. The range of the ultrasound flow measurement of nine cardiac cycles (CC) is shown

The flow curves in Fig. 6a show the results of the PC-MRI measurements of the volunteers representing the healthy population [3, 38]. Since their heart rates varied, data was adjusted to one CC. The cervical flow was measured in ml/min with the flow direction from cranial to caudal defined as positive and the reverse flow as negative. The maximum PC-MRI flow was 122.86 ml/min in the caudal and 77.86 ml/min in the cranial direction (Table 2). Furthermore, the SV were calculated and compared to physiological SVs in the spinal canal reported in literature. The SV was computed by the integration of the mean flow and results per CC in 0.385 ml for the PC-MRI measurements (Table 3).

**Table 2:** Mean bidirectional maxima CSF flow in the spinal canal divided into in vitro and in vivo (PC-MRI) data with maximal and minimal deviations

Measurement	Flow direction	Mean in ml/min	Deviations in ml/min
In vivo	${Q}_{{{cranial-caudal}}}$	122.82	+ 29.61
			− 38.27
	${Q}_{{{caudal-cranial}}}$	− 77.86	+ 22.02
			− 21.28
In vitro	${Q}_{{{cranial-caudal}}}$	133.60	+ 10.31
			− 11.67
	${Q}_{{{caudal-cranial}}}$	− 68.01	+ 14.43
			− 21.09

**Table 3:** Spinal stroke volume (SV) in ml per cardiac cycle (CC)

	Stroke volume	References
In vitro	0.312	Integration of flow
In vivo	0.385	Integration of flow
Physiological	0.272–0.699	[14, 38, 39, 43–45]

### In vitro flow—ultrasound flow sensor

The resistance, compliance and blood dynamics in the system influence the results of the in vitro measurement. Therefore, the parameter setup was not changed during the flow and pressure recordings. The AV flow is shown in Fig. 4 and the compliance volume and distribution in Table 1.

The in vitro cervical flow was measured over nine CCs and is displayed in Fig. 6b, showing the range of the flow recorded by the ultrasound sound meter. The maximum of the mean in vitro measurement was 133.60 ml/min in the caudal and 68.01 ml/min in the cranial direction (Table 2) with a mean SV of 0.312 ml/CC (Table 3). The point in time at which the flow in the cranial direction was maximal (the minimums of the plots in Fig. 6), did not coincide for the two measuring methods. Taking the maximum caudal flow as the start and end (0 and 100%, respectively), the maximum in vitro flow towards the cranium measured occurred at around 25% of the CC, whereas the in vivo maximum was at approximately 63%, the latter varying by about 10% with the individual data.

### Pressure curves

**Fig. 7:**
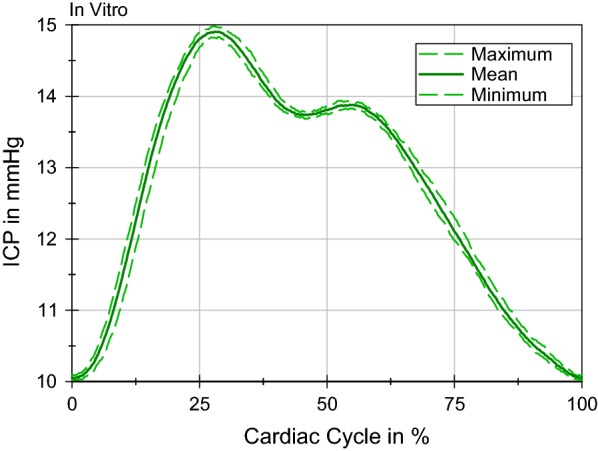
In vitro ICP measured with the tip-catheter sensor in the ventricular system. The range of the same nine cardiac cycles as Fig. 6b is shown

The ICP, measured inside the ventricular system over one CC in a supine position is shown in Fig. 7. There are three lines: The two dashed lines represent the maximal and minimal pressure progression and the continuous line, the mean ICP. The arithmetic mean ICP value over nine CCs was 12.68 mmHg. The maximum ICP was 14.98 mmHg and the minimum was 10.02 mmHg. Furthermore, two pressure peaks were identified with a ratio of (P2:P1) 0.792 and a mean wave amplitude (MWA) of the first pressure peak at 4.86 mmHg. Control measurements with the other two pressure sensors (Cistern, Cranial SAS) showed no significant deviations.

## Discussion

### Flow measurements

**Fig. 8:**
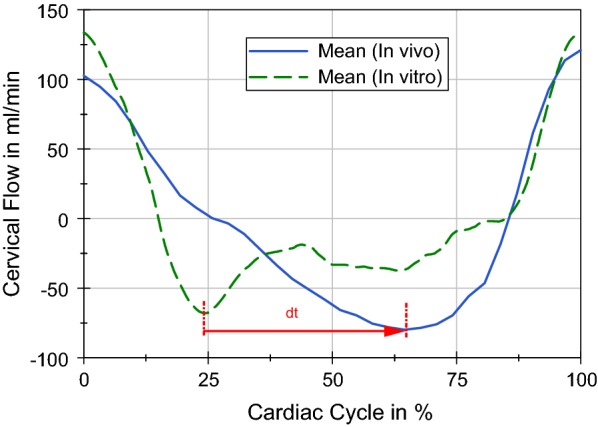
Mean spinal CSF flows. This graph depicts the comparison of mean spinal CSF flows of the in vitro measurement (green) and the PC-MRI data of nine subjects (blue) derived from Fig. 6a, b

The extreme values of the in vitro flow measurements are in good agreement with the PC-MRI data as they were within the tolerance range of the PC-MRI measurements. Furthermore, other research groups support the recorded flow measurements with similar flow values in both directions [39, 40]. Additionally, both curves showed a typical steep rise during systole. Moreover, the occurrence of minimal flow in the in vitro measurement developed ahead of the PC-MRI flow minimum (dt in Fig. 8). This could occur due to a return oscillation or reflection of the arterial pulse wave, since only a static compliance, represented by the air in the compliance chambers, has been taken into account so far in the experimental setup. However, the brain and the tissue surrounding the craniospinal system has viscoelastic properties that require a time-dependent or dynamic compliance [15, 32, 41, 42].

In addition, the spinal SV of the phantom (0.312 ml/CC) was in the same range as the PC-MRI measurements of healthy volunteers (0.385 ml/CC), defining a physiological range for the SV from 0.272 to 0.699 ml/CC [14, 38, 39, 43–45] (Table 3).

### Pressure measurements

The in vitro results of ICP are compared with literature values as no ICP measurements have been performed on the healthy population undergoing PC-MRI measurements. The mean ICP in a horizontal position was 12.68 mmHg and is, thus, in a physiological range [6, 46–49]. Considering there is a lack of data on the maximum ICP amplitude, because invasive ICP recordings are not performed on healthy individuals, the measurement of the maximum amplitude cannot be classified as physiological. However, the MWA of NPH patients, for instance, is used to categorize patients into two groups: Those who respond to the placement of a shunt and those who do not [50, 51]. The pulse wave amplitude in the in vitro model was below 5 mmHg and is, therefore, still in a similar value range as the data reported. An MWA above 5 mmHg would be considered conspicuous. Furthermore, an additional dynamic compliance would further decrease the MWA. Finally, the pressure curves demonstrated the stability of the model and the pressure measurement, since the maximum and minimum curves deviated by only 0.148 mmHg in the extreme values over various CCs.

### Limitations and prospects

The validation of the model presented can only be applied in a supine position, because all measurements (in vivo and in vitro) were performed in this position. If the position is changed to upright, the compliance values and divisions must be adjusted, because they vary with patient position [35, 52] and influence the CSF dynamics. A pressure sensor can be added to the end of the spinal canal to investigate pressure dynamics with changing hydrostatics. Furthermore, we hypothesize that the craniospinal compliance is time-dependent due to its viscoelastic properties or breathing and, thus, has to be considered when modeling the CSF dynamics. Regarding the in vitro model, we expect the mean ICP wave amplitude to decrease and the time of the maximum spinal CSF flow in a cranial direction to shift when the dynamic compliance is incorporated into the model. Moreover, our measurements have shown that the pressure and flow curves provide results in the physiological range with a higher spinal compliance compared to the cranial compartment. However, this division is still being controversially discussed [31, 34–36] and should be examined more closely in future measurements, specifically in terms of dynamic values and distribution.

In addition, it should be noted that the young and healthy volunteers (PC-MRI) were limited to nine subjects. However, they represent a healthy population [3]. Furthermore, the flow in the aqueduct should be measured, because its pulsatility and SV can be further indicators of NPH [53–56]. Another technical limitation is related to the flow measurement using an ultrasonic sensor. Although this method has the advantage of contactless measurements and deviations of $+/-\,6$ ml/min based on its technical specifications, due to the strongly pulsating flow, extrema show deviations of up to 21.09 ml/min, while the mean values of flows over one CC only vary by 8.79 ml/min.

The test bench can be extended due to the modular setup, which allows the addition of a variety of applications (e.g. breathing). In addition to investigating the pathogenesis of NPH, parameter analysis on spontaneous intracranial hypotension (a leak in the spinal canal) or syringomyelia (a cavity in the spinal canal) could be conducted in in vitro studies. Moreover, aging-related changes, such as a reduced blood flow, an AV delay [14], arterial stiffness [57], an increased resistance to outflow [10, 11] or a parenchymal liquefaction [58], can be analyzed. Furthermore, the test bench can be used to test alternative therapies and implants.

## Conclusion

In conclusion, the in vitro results showed a good correlation with in vivo data and literature values regarding ICP and SVs. However, it emerged that the dynamic compliance cannot be neglected, especially for the analysis of the effects of high-pressure gradients and the strains on viscoelastic tissue. By integrating a dynamic compliance, known age-related or pathological changes in viscoelastic cerebrospinal tissue [58, 59] could be investigated. The main goals of our ongoing research are the sensitivity analyses of the blood dynamics by exchanging the cam disk or the frequency, the (dynamic) compliance behavior, the changed resistances (stenosis), the influence of hydrostatics and the integration of production and an adjustable absorption.

## Abbreviations

AV: arteriovenous
CC: cardiac cycle
CSF: cerebrospinal fluid
ICP: intracranial pressure
MWA: mean wave amplitude
NI: national instruments
NPH: normal pressure hydrocephalus
p: pressure
PMMA: polymethylmethacrylate
PC-MRI: phase-contrast magnetic resonance imaging
SAS: subarachnoid space
SV: stroke volume
V: volume
